# The Burden of Hypertension and Prehypertension in a Community Health Centre of Haryana

**DOI:** 10.7759/cureus.33569

**Published:** 2023-01-09

**Authors:** Pooja Sindwani, Seema Sharma, Aftab Ahmad, Amit Kumar, Sapna Dalal, Piyush Jain

**Affiliations:** 1 Community Medicine, Teerthanker Mahaveer Medical College and Research Center, Moradabad, IND; 2 Community Medicine, Maharaja Agrasen Medical College, Hisar, IND; 3 Community Medicine, Adesh Medical College and Hospital, Shahbad, IND; 4 Respiratory Medicine, Teerthanker Mahaveer Medical College and Research Center, Moradabad, IND

**Keywords:** north india, non-communicable disease, chc, pre-hypertension, hypertension

## Abstract

Background

Hypertension (HTN) is endemic in India and it is considered a public health challenge in both economically developed and developing nations. Unfortunately, despite its high prevalence, its awareness, treatment, and control status are low in urban as well as rural Indian populations.

Objectives

To determine the burden of hypertension and prehypertension in a Community Health Center (CHC) and to find the association of hypertension with the age group and sex of study subjects.

Methodology

A cross-sectional study was carried out among 713 patients of age 20 years and above attending the Out Patient Department (OPD) of the Community Health Centre (CHC), Barwala in the Hisar district of Haryana. JNC 7 classification of blood pressure was used to diagnose hypertension. The collected data was analysed using the Statistical Package for Social Sciences (SPSS) version 20.0. Appropriate statistical tests were used.

Result

Out of the total patients enrolled, 200 (28.1%) were found to be hypertensives. The burden of prehypertension was further observed to be 28.1%. About (61.6%) of OPD patients were female. Among hypertensive patients, nearly half (48.8%) were in the age group of 60-80 years whereas the majority of the hypertensives (56%) were females.

Conclusion

In our study, more than 50 percent of OPD patients were found to have hypertension and pre-hypertension. Health-seeking behavior was more among females.

## Introduction

Hypertension (HTN), among other non-communicable diseases, is endemic in India [1]. It is considered a public health challenge in both, economically developed and developing nations [2]. The overall occurrence of high blood pressure is almost similar between both men and women but differs with age [3]. Existing literature has shown that hypertension is more common in men less than 45 years of age, whereas it affects women more than men above 65 years of age [4,5]. It is hypothesized that the hormonal changes in females that occur due to menopause are responsible for this difference. HTN is known as the “silent killer” because it typically has no warning signs or symptoms, and many people are not even aware of it [2]. It is known that approximately 70% of people who have their first heart attack already have HTN. About 80% of people who had an episode of stroke for the first time have high blood pressure [4]. One of the global targets for non-communicable diseases is to reduce the prevalence of hypertension by 25% by 2025 [6]. The lifestyle changes which have affected people in urban areas more than in rural areas have led to an increase in the prevalence of hypertension. This predisposes people to increased incidence of mortality and morbidity due to coronary, cerebrovascular, and kidney disease. Every fourth individual in India aged above 18 years has been reported to have raised blood pressure, and the prevalence is expected to increase further. This study was conducted to determine the burden of hypertension and prehypertension in a Community Health Center (CHC), and to find the association of hypertension with the age group and sex of study subjects. Such studies are needed from time to time so as to add data to the existing literature and help in planning preventive strategies to reduce the burden. This will further help in the formulation of cost-effective interventions for people with the established disease for a significant reduction in morbidity and mortality arising due to complications of hypertension.

## Materials and methods

A cross-sectional study was carried out among 713 patients aged 20 years and above attending the out-patient department (OPD) of the Community Health Center (CHC), Barwala in the Hisar district of Haryana, in North India. CHC Barwala caters to 8 Primary Health Centers namely Barwala, Dhansu, Agroha, Landhri, Uklana, Pabra, Daulatpur, and Hasangarh, and covers a population of approximately 157,981. The total OPD census was 8640 during the period of data collection. The study was conducted from March 2019 to August 2019. A formula for cross-sectional study for calculation of sample size was used before proceeding with the study. Assumptions made for the calculation of sample size were based on NFHS 4 data with an expected prevalence of hypertension to be 22.4%, an alpha error of 5%, and an acceptable error in the measurement to be 3%. Study subjects were initially chosen by selecting the first patient randomly from one to five by lottery method then every 5th study subject was chosen by systematic random sampling. All patients aged 20 years & above including pregnant females who attended OPD of CHC, Barwala, and willing to participate were included in the study. However, patients with any acute severe illness and any women with pregnancy-induced hypertension were excluded from the study. Variables such as name, age, sex, and BP readings were recorded in a register. JNC 7 classification of blood pressure for adults was used to classify the BP readings into normal, prehypertension, and hypertension. Cut-off values for BP were taken as patients with either systolic BP ≥ 140 or diastolic BP ≥ 90 or both were considered hypertensive. Patients with either systolic BP between 120-139 mmHg or diastolic BP between 80-89mmHg or both were considered as prehypertensive. Blood pressure was measured by using an aneroid sphygmomanometer. Participants were made to sit quietly for at least 5 minutes with the back supported by the chair backrest, with feet on the floor and arms supported at heart level. The BP measurements were performed on the left arm after resting the arm on the table at the level of the heart. Systolic blood pressure (SBP) was noted at the point when the first of two or more Korotkoff sounds heard (onset of phase 1) and the disappearance of Korotkoff sound (onset of phase 5) was used to define diastolic blood pressure (DBP). Patients with either systolic BP ≥ 140 or diastolic BP ≥ 90 or both were considered as hypertensive. BP was measured by a primary care physician. The average of the 2 blood pressure readings, as recommended by JNC 7, was taken. The collected data were analyzed using the Statistical Package for Social Sciences (SPSS) version 20.0. Appropriate statistics (mean, median, standard deviation, Chi-square test) were applied to draw relevant inferences. A p-value of <0.05 was considered statistically significant. Ethical clearance was obtained from the Institutional Review Board (IRB) of Maharaja Agrasen Medical College, Agroha vide reference no. MAMC/Pharma/IEC/19/14 of Institutional Ethics Committee for Human Research before starting the study. Informed and written consent was obtained before enrolling the patients.

## Results

**Figure 1:**
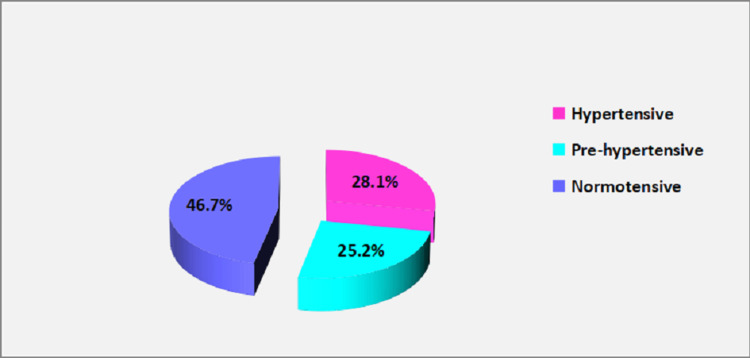
Burden of hypertension among OPD patients

Among 713 patients attending the OPD, 200 (28.1%) patients were found to be hypertensive and 513 (71.9%) patients as non-hypertensive (180 as prehypertensive and 333 as normotensive). The overall burden of hypertension and prehypertension was observed to be 28.1% and 25.2% respectively (Figure 1).

The mean age of hypertensive and non-hypertensive patients was 51.62 ±12.82 and 42.36 ± 12.61 respectively, and this difference between the mean age of the two groups was found to be statistically significant (p = 0.001). The burden of hypertension was maximum (48.8%) in the age group of 60-80 years. The observed difference between increasing age and the burden of hypertension was found to be statistically significant (p<0.001). Among males and females, the burden of hypertension was found to be 32.1% and 25.5% respectively. The observed difference between sex and the burden of hypertension was not found to be significant (p = 0.056). The mean systolic BP of hypertensive patients and non-hypertensive patients were 152.35 ±13.85 mmHg and 110.75±13.49 mmHg respectively, and this difference between mean systolic BP of the two groups was found to be statistically significant (p = 0.001). The mean diastolic BP of hypertensive patients and non-hypertensive patients were 97.09 ± 10.13 mmHg and 70.64 ± 9.08 mmHg respectively, and this difference between the mean diastolic BP of the two groups was found to be statistically significant (p = 0.001) (Table 1).

**Table 1:** Association of hypertension with different demographic parameters (n=713)

Variable	HTN	Non-HTN	p-value
Age (mean+SD)	51.62 ± 12.82	42.36 ± 12.61	0.001
Age groups n (%)			
20 – 39 years	27 (11)	218 (89)	0.001
40 – 59 years	111 (32.6)	230 (67.4)	
60 – 80 years	62 (48.8)	65 (51.2)	
Sex n (%)			
Male	88 (32.1)	186 (67.8)	0.056
Female	112 (25.5)	327 (74.5)	
Systolic BP (mean+SD)	152.35 ± 13.85	110.75 ± 13.49	0.001
Diastolic BP (mean+SD)	97.09 ± 10.13	70.64 ± 9.08	0.001

Out of a total of 713 OPD patients, a majority (341) of them were in the age group of 40-59 years. The majority of OPD patients (61.6%) were females. The observed difference between age group and sex was found to be statistically significant (p=0.01) (Table 2). This signifies that the health-seeking behavior was more among females of all age groups as compared to males.

**Table 2:** Association of age group with the sex of OPD patients (n=713)

Age Groups(in years)	Male	Female	p-value
	n (%)	n (%)	
20 - 39	76 (31.0)	169 (69.0)	0.01
40 - 59	141 (41.3)	200 (58.7)	
60 - 80	62 (44.9)	65 (55.1)	
Total	274 (38.4)	439 (61.6)	

## Discussion

A meta-analysis conducted in 2014 by Anchala et al. [7] found a similar prevalence of hypertension (29.8%). A slightly higher prevalence was found in studies carried out by Dhungana et al. [8] (32.5%) in municipalities of Kathmandu, Nepal in 2016, and Singh et al. [9] (32.9%) in urban Varanasi in 2017. A much higher prevalence (63.7%) was found in a study conducted by Rouf et al. in the adult population of Block Hazratbal, Srinagar in 2018 [10].

Other studies which reported a higher prevalence of hypertension were those carried by Awino et al. [11] (36.9%) among adult patients attending Yala Sub-County Hospital, Siaya County (Kenya) in 2016, Kafle RC et al. [12] (41.55%) in a rural community of western Nepal 2018, Banerjee et al. [13] (42.0%) in the slums of Kolkata in 2015, and Chandra et al. [14] (48.0%) in the field practice area of Rajiv Gandhi Institute of Medical Sciences, Kadapa in 2011.

A much lower prevalence was also found in some studies carried out by Gudina et al. [15] (13.2%) in southwest Ethiopia in 2013, Kishore et al. [16] (14.1%) in Rural Delhi in 2016, Sharma et al. [17] (14.2%) in a rural area of Madhya Pradesh in 2015, and Islam et al. [18] (23.7%) in the urban area of Bangladesh in 2015. The observed difference might be due to different geographical areas. Various studies have reported a wide range of prevalence of hypertension. Kearney et al. [19] analysed worldwide data and also concluded the varied prevalence of hypertension with the lowest prevalence in rural India (3.4% in men and 6.8% in women) and the highest prevalence in Poland (68.9% in men and 72.5% in women).

Age group-wise comparison of hypertension

Age is known to be an important risk factor for hypertension. As age advances, so did the prevalence of hypertension among both sexes. Similar findings were reported in our study where hypertensive were older (51.62 ±12.82) as compared to non-hypertensive (42.36 ±12.61) which was found to be statistically significant at p<0.001. This finding was consistent with a study done by Siddiqui et al. [20] in which the mean age of hypertensives (38.7±16.5) was significantly greater (p<0.001) than normotensives (29.2±13.6).

In our study, the number of hypertensive patients increases with increasing age groups. In lower age groups (20-39 years), 11% of patients reported having hypertension, followed by 32.6% and 48.8% in 40-59 years and 60-80 years, respectively, which was statistically significant with a p-value <0.001. Kafle et al. [12], Dhungana et al. [8], and Mosha et al. [21] also reported that the prevalence of hypertension increases with the increasing age group. This finding was similar to a study done by Jindal et al. [22] which also reported a higher prevalence of hypertension among individuals greater than 40 years of age. This finding can be explained by the fact that with increasing age, the aorta and walls of arteries get stiffened, and this contributes to the high prevalence of hypertension in older age groups [23].

Sex-wise comparison of hypertension

In the current study, among 713 OPD patients, 32.1% of males and 25.5% of females are reported to have HTN. Almost similar prevalence was reported by a study carried out by Dhungana et al*.* This study reported a prevalence of hypertension among males to be 38.4%, and a 28.4% prevalence of HTN was reported among females. [8] Similarly in this study males were reported to have a higher prevalence of HTN as compared to females, similar to other studies conducted by Singh et al. [9] (male: 40.9%, female: 26.0%) and Awino et al. [11] (male 38.9%, female 35.7%). This gender disparity in hypertension prevalence could be explained partially due to biological sex differences and partially due to behavioral and risk factors of hypertension like smoking, alcohol consumption, or physical activity which are either less or absent in women, as reported by Singh et al. [9]. In this present study, among all age groups, 61.6% of OPD patients were females as compared to 38.4% of males. This suggests that the health-seeking behavior was more among females of all age groups as compared to males. Few studies also supported the finding that women are more interested in healthcare services utilization and also more likely to express their poor health leading to having a high awareness of the condition. This results in having better health in women as compared to men [24,25,26].

Despite our efforts, this study had some limitations which have been accounted for while analyzing and reporting the findings of the study. One of the limitations of the study was that the patients were enrolled in a government-operated healthcare facility. This may not reflect the actual burden in the population. This was primarily due to operational feasibility.

## Conclusions

The present study confirmed a high burden of hypertension in the study area. It was also found that the burden of hypertension increased significantly with advancing age. Therefore, it is necessary that effective preventive and control measures are implemented with a special focus on the geriatric age group. Further, one-fourth of people were categorized as pre-hypertensives and hence, control strategies for the prevention of hypertension should be implemented with a special focus on preventing the progression of prehypertensives to hypertensives. Health camps should be routinely organized.
